# Respiratory outcomes with the use of a lower custom fit genioglossal‐effecting oral appliance

**DOI:** 10.1002/cre2.254

**Published:** 2020-01-06

**Authors:** Dena P. Garner, Jensine Lamira

**Affiliations:** ^1^ Department of Health and Human Performance, the Citadel The Citadel Charleston South Carolina

**Keywords:** exercise, genioglossus, oral appliance, respiratory rate, ventilation

## Abstract

**Objective:**

Sleep apnea research cites that an oral appliance, which places the mandible in a more forward position and the genioglossus (tongue muscle) on the floor of the mouth, improves aspects of the pharyngeal opening. Exercise science research has cited improvements in airway dynamics and physiological variables with oral appliance use during exercise. Thus, the purpose of this study was to design an oral appliance that would act on the genioglossus and determine if there were effects on respiratory parameters while exercising.

**Materials and methods:**

Seventeen healthy subjects ages 18–43 participated in this study. Prior to the exercise protocol, the order of the oral applicance (OA) or no oral appliance (no OA) condition was randomly assigned to subjects, with subjects completing both conditions. Respiratory parameters (respiratory rate, ventilation, oxygen, and carbon dioxide) were measured between conditions while subjects ran for 10 min at steady state.

**Results:**

The results demonstrated that both respiratory rate (25.97 BPM, OA and 28.35 BPM, no OA) and ventilation (47.66 l/min, OA and 50.34 l/min, No OA) were significantly lowered (*p* < .01) in the OA condition. There were no differences in carbon dioxide (1.89 l/min, no OA and 1.88 l/min, OA) or oxygen outcomes (2.17 l/min, no OA and 2.17 l/min OA).

**Discussion:**

The outcomes from this study suggest that the design of the oral appliance elicits an effect on the genioglossus, thereby resulting in lowered respiratory rate and ventilation with no negative effects on oxygen uptake during exercise.

## 1 INTRODUCTION

The exploration of the use of an intraoral appliance on human performance is not novel. In recent years, oral appliances have gained traction in the field of exercise physiology with findings that support varied physiological improvements during exercise to include changes in lactate, cortisol, power output, and lowered respiratory rates (Arent, McKenna, & Golem, 2010; Bailey et al., 2015; Busca, Morales, Solana‐Tramunt, Miró, & García, 2016; Dudgeon, Buchanan, Strickland, Garner, & Scheett, 2017; Dunn‐Lewis et al., 2012; Durante‐Pereira et al., 2008; Ebben, Leigh, & Geiser, 2008; Garner & McDivitt, 2009; Morales, Busca, Solana‐Tramunt, & Miro, 2015). These changes in respiratory parameters with an oral appliance may be related to outcomes cited in sleep apnea research, specifically assessing tongue and mandibular placement. Research has cited that the placement of the tongue and mandible and the application of a mandibular appliance will affect airway openings. Thus, understanding the importance of its role and implication of the genioglossus is critical to the understanding of how these mouthpieces may effect a change.

Gilbert and colleagues cite that the genioglossus is referred to as a muscular hydrostat, meaning that it is a muscle that creates movement and also serves as the support for that movement ( Gilbert, Napadow, Gaige, & Wedeen 2007). It does this because the muscle fibers of the genioglossus include intrinsic and extrinsic fibers that are perpendicular and parallel within the organ. This is important, as the role of the extrinsic muscles in the tongue was thought to solely manipulate the tongue position, whereas the intrinsic muscles served to alter its shape. Yet more research has discovered that extrinsic and intrinsic tongue muscles operate in concert to elicit an effect on physiological functions, such as swallowing and respiration, which has been confirmed with the measurement of airflow via magnetic resonance imaging tagging (Cheng, Butler, Gandevia, & Bilston, 2008; Gilbert, Napadow, Gaige, & Wedeen, 2007). Magnetic resonance imaging outcomes cited minimal movement of the other tissues in the mouth, thereby suggesting that the tongue is the primary active dilator of the upper airway (Cheng et al., 2008; Saboisky, Luu, Butler, & Gandevia, 2015). Thus, when the genioglossus is stimulated, it results in the dilation of the airway, with increases of 33 to 284% of the hypopharyngeal airway due to activation of the genioglossus (Fregosi & Ludlow, 2014; Mann, Burnett, Cornell, & Ludlow, 2002). Driving this activation of the tongue is a complex integration of premotor networks via the hypoglossal motor nucleus supporting airway patency and other functions such as swallowing and speech (Saboisky et al., 2006).

The research regarding tongue placement in healthy populations during relaxed/resting states varies with supine to upright protocols. Hiyama and colleagues examined muscular movements with electromyography (EMG) activation as it correlates with activity of the jaw opening and closing, finding increased genioglossal EMG activity with the jaw closing phase (Hiyama, Iwamoto, Ono, Ishiwata, & Kurodo, 2000). As it relates to tongue placement on physiological functions, Schmidt and colleagues assessed differences in heart rate when the tongue was on the palate or the floor of the mouth. The placement of the tongue on the roof of the mouth increased muscle activation of the temporalis and suprahyoid muscles, which in turn affects physiological functions such as heart rate variability (Hiyama et al., 2000; Schmidt, Carlson, Usery, & Quevedo, 2009; Takahashi, Kuribayashi, Ono, Ishiwata, & Kurodo, 2005; Valdes, Astaburuaga, Falace, Ramirez, & Manns, 2014). Interestingly, di Vico and colleagues assessed changes in tongue placement and effect on isometric knee extension exercises, citing changes in force outputs with differences in tongue position. However, the authors noted that the study was preliminary, and future research should investigate tongue placement as it affects muscular output in other limbs (di Vico, Ardigo, Salernitano, Chamari, & Padulo, 2013). In a tightly controlled study, Sabiosky and colleagues cited decreases in tongue protrusion force once the tongue protruded beyond the incisors (Saboisky et al., 2015). Others have cited that the changes in respiratory rate may be due to position of the tongue as well as activation of the tongue muscle. It has been hypothesized that the improvements in respiratory rate with use of a specific mandibular oral appliance may be due to the contraction of the genioglossus, whereas others noted that the positive respiratory outcomes could be attributed to pursed lipped breathing, which involves changes in tongue placement (Francis & Brasher, 1991; Garner, 2015).

Thus, based on the hypothesis of genioglossus placement, as elicited by the use of an oral appliance and subsequent positive effects on performance, the purpose of this research was to research the effect of a mandibular device designed to produce a change on the genioglossus.

## 2 MATERIALS AND METHODS

Healthy, physically fit subjects over the age of 18 years were invited to partake in this study (Table 1). The institution's internal review board approved this study, and all subjects completed a consent form prior to participating. All research was completed in accordance with legal requirements of the United States, which includes an approval of our institution's ethical committees for human research (IRB 12‐1315).

**Table 1:** Demographics of subjects

Age, mean (SD)	25.71 (9.03)	
Gender, *N* (%)	Male	13 (76.5%)
	Female	4 (23.5%)

**Figure 1:**
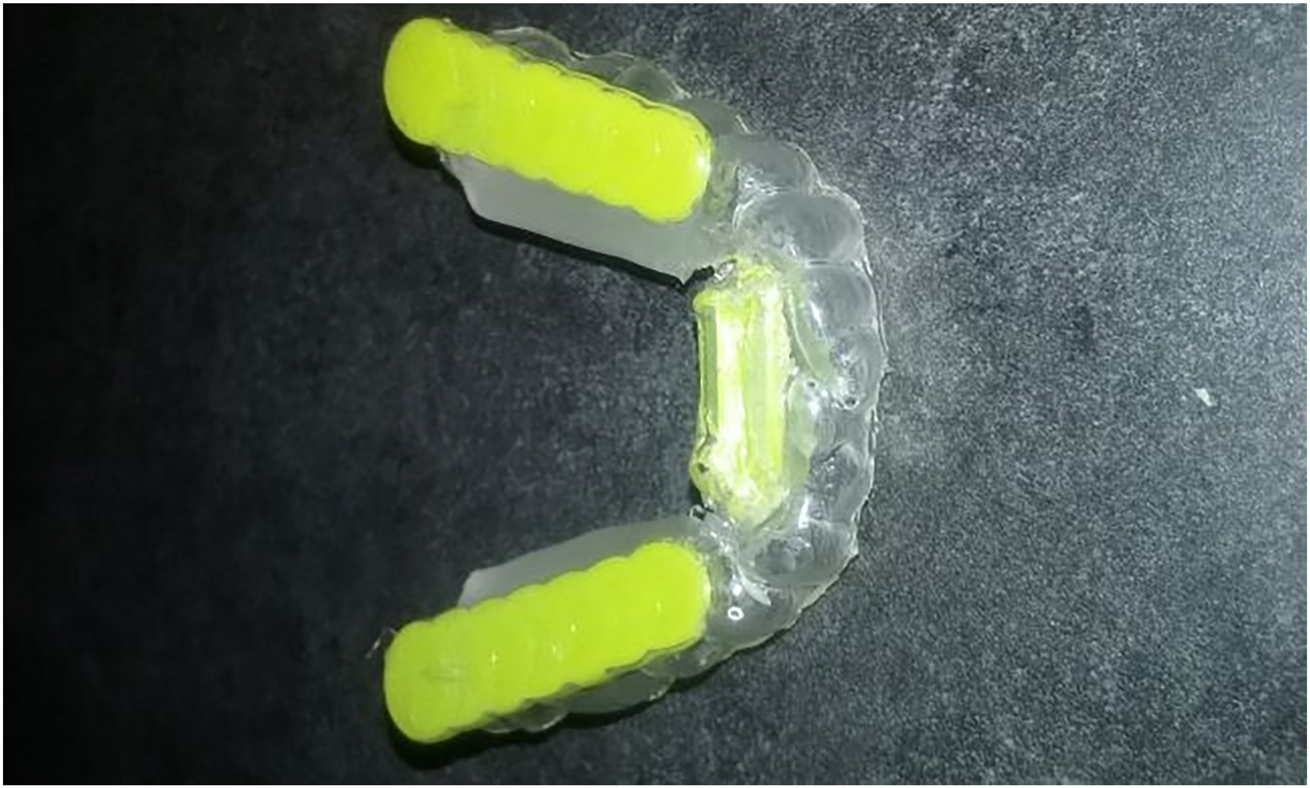
Top view of the custom fit mandibular oral appliance

A lower custom oral appliance was designed for subjects, which resulted in tongue placement on the floor of the mouth and protrusion of the tongue beyond the incisors being prevented and subsequently measured effect on respiratory parameters during steady‐state exercise (Figure 1).

Before exercise testing began, the researchers took a mandibular impression of each subject using ACU‐flow hydrophilic impression material. Denstone Golden was used to cast the molds, which were then utilized to create the mandibular mouthpieces on site. Each oral appliance was made using a Biostar Scan/Biostar V (Great Lakes Orthodontics, LTD Tonawanda, NY 14150). The mandibular devices, which had been pilot tested prior to the study to determine efficacy, were designed to manipulate the genioglossus in a forward position, with an interior attachment to maintain the placement of the tongue on the floor of the mouth during testing. In addition, bite plates were fabricated and embedded into the custom oral appliance to encourage subjects to bite down and push the tongue under the interior lower attachment.

The subjects completed a 5‐min warm up at 5.0 mph, 3‐min cool down at 3.0 mph, 10‐min steady state at 6.0 mph, and 3‐ and 2‐min cool down at 3.0 and 2.0 mph, respectively. Each subject performed the testing on the same day with 5 min between testing to allow for rest and heart rates to return to baseline. Because the protocol was a short, steady‐state protocol and not rigorous for a healthy group of participants, testing on the same day with adequate rest between sessions should not affect the outcomes. However, to minimize any of these effects, the use of the oral appliance and no oral appliance condition was randomized to account for muscles warmed by the first run.

All subjects were fit with a heart rate monitor (Polar Heart rate monitor, Polar Electro Inc., Lake Success, NY). To measure respiratory parameters, a metabolic cart (TrueMax 2400 Pravo Medics, Sandy, UT, USA) was utilized to measure differences in oxygen (VO_2_), carbon dioxide (VCO_2_), respiratory rate (RR), and ventilation (Ve). Each subject wore a mask that covered his/her nose and mouth, with the nose pinched together by a clip, which allowed normal breathing patterns. The masks were inspected prior and during testing to ensure proper fit to confirm no gas leaking occurred during testing.

The subjects served as their controls, and a within‐subjects design was conducted. All data were entered into Microsoft Excel for management and exported to IBM SPSS 25.0 (IBM Corporation, Armonk, New York) for statistical analysis. Comparison of VO_2_, VCO_2_, RR, and Ve was done using repeated measures analysis of variance and a paired *t* test between conditions. Percent change scores between group means were calculated using the following formula: (μ2 − μ1/μ1) × 100. Statistical significance was set at *p* < .05. All data are presented as mean ± standard deviation (Table 2).

**Table 2:** Respiratory parameters averaged over 10‐min run during steady‐state exercise (*N* = 17)

Parameter	NO MP, Mean (SD)	MP, Mean (SD)	*p* value (two‐tailed)
RR (BPM)	28.35 (7.24)	25.97 (7.03)	.000*
Ve (l/min)	50.64 (12.18)	47.66 (12.92)	.000*
O_2_ (l/min)	2.17 (0.57)	2.17 (0.59)	.731
CO_2_ (l/min)	1.89 (0.49)	1.88 (0.53)	.066

Abbreviations: CO_2_,carbon dioxide exhalation; MP, mouthpiece condition; No MP, no mouthpiece condition; O_2_,oxygen uptake; RR, respiratory rate; Ve, ventilation.

* *p* < .01

## 3 RESULTS

**Figure 2:**
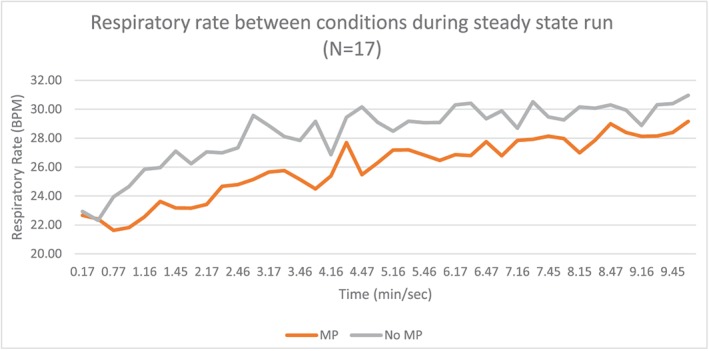
Respiratory rate between no mouthpiece (no MP) and mouthpiece (MP) conditions averaged across subjects during the 10‐min steady‐state run

**Figure 3:**
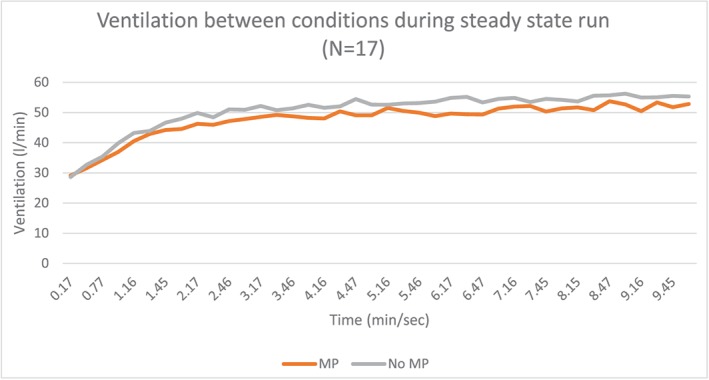
Ventilation between no mouthpiece (no MP) and mouthpiece (MP) conditions averaged across subjects during the 10‐min steady‐state run

**Figure 4:**
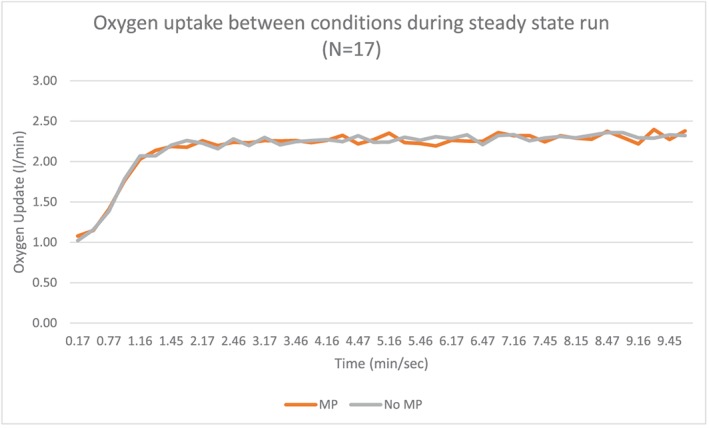
Oxygen uptake between no mouthpiece (no MP) and mouthpiece (MP) conditions averaged across subjects during the 10‐min steady‐state run

**Figure 5:**
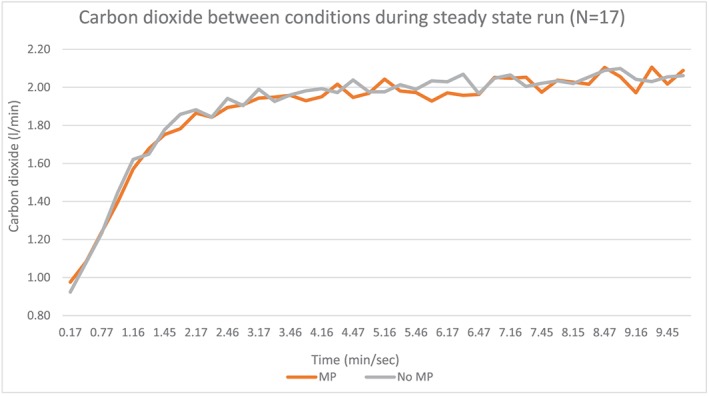
Carbon dioxide exhalation between no mouthpiece (no MP) and mouthpiece (MP) conditions averaged across subjects during the 10‐min steady‐state run

Seventeen subjects with an average age of 25.7 years (standard deviation 9.03) completed the study (Table 1). Data analyzed during the 10‐min steady‐state run between conditions resulted in significant decrease (*p* < .01) in respiratory rate in those wearing the oral appliance (OA) versus a no oral appliance (no OA) condition (OA, 25.97 [7.03] BPM and no OA, 28.35 (7.24) BPM). In addition, averaging respiratory rates across the 10‐min period was also significant (*p* = .01; see Figure 2 and Table 2). Ventilation (Ve) was also significantly lowered in OA versus no OA condition as averaged between subjects and over the 10‐min run (see Figure 3 and Table 2). There were no differences in volume of oxygen uptake (l/min) averaged and across the 10‐min period (see Figure 4 and Table 2). There were no significant differences in carbon dioxide (l/min) averaged across the 10‐min run, but it trended higher during the first 7 min of the run with the no OA condition, but this dissipated during the last 3 min of the run (see Figure 5 and Table 2).

## 4 DISCUSSION

The results demonstrate that the use of a lowered custom fit oral appliance supports what other studies have found related to respiratory function and use of an oral appliance during aerobic steady‐state exercise, finding a positive impact on respiratory parameters with oral appliance use (Bailey et al., 2015; Bourdin et al., 2006; Francis & Brasher, 1991; Garner, 2015; Garner, Dudgeon, Scheett, & McDivitt, 2011; Gebauer, Williamson, Wallman, & Dawson, 2011). However, until this present study, the cause of these differences in respiratory parameters has not been fully clarified. Garner et al. and Bailey et al. cited the mouthpiece design as a potential cause of the decrease respiratory/ventilation rates within their subjects (Bailey et al., 2015; Garner, 2015). Thus, based on the previous findings by others in this field, one aspect of the current oral appliance constructed for this study sought to elicit pursed lipped breathing with a conformational change in the tongue position.

In addition, in developing this mouthpiece, understanding the design of oral appliances utilized in sleep apnea medicine was also considered. In those studies, researchers cited that an oral appliance affects tongue/genioglossus placement and subsequently produces the positive opening of the airway (Gale et al., 2000; Johal, Gill, Ferman, & McLaughlin, 2007; Kyung, Park, & Pae, 2005). The tongue consists of four extrinsic and four intrinsic muscles innervated by the hypoglossal nerve, with the genioglossus being one of the extrinsic muscles that protracts the tongue and affects airway dynamics (Bailey, Huang, & Fregosi, 2006; Fregosi & Ludlow, 2014; Hiyama et al., 2000; Mann et al., 2002). The jaw and tongue muscle play a significant role in the pharyngeal airway opening, with the contraction of the genioglossus as it pulls the base of the tongue down and forward, supporting an enhanced pharyngeal airway (Bailey et al., 2006; Mann et al., 2002; Miller, 2002; Saboisky et al., 2006). Thus, based on these findings, the design of the oral appliance used in this study sought to stimulate the genioglossus, contracting it, and placing it on the floor of the mouth during exercise and then determine if there was a subsequent effect on respiratory parameters.

Although this current study did not measure EMG activity of the genioglossus, subjects were told to keep their tongue under the front aspect of the oral appliance during the protocol and breathe through their mouths, which placed the tongue at the base of the oral appliance in a more forward position. The results of this study support further testing to determine if there is a correlation between the stimulation of the genioglossus, enhanced airway, and effect on respiratory parameters. However, in studying the stimulation of the genioglossus, there are challenges. Currently, best practices in measuring genioglossal activity utilize stainless steel Teflon‐coated wire electrodes inserted under the chin and into the genioglossal muscle. When adding an oral appliance to the protocol during exercise, this can be difficult for both the researcher and subject due to the EMG noise associated with the exercise movements and the potential pain associated with needle insertion. Thus, future collaborative research must include the various pieces of this research to support or refute our hypothesis that the genioglossus muscle is being stimulated with the current designed oral appliance in healthy subjects. The positive outcomes with respiratory rate and ventilation were needed as the first step in understanding if the currently designed oral appliance was effective.

The goal in choosing this protocol was to compare/contrast to prior studies, which had also utilized a steady‐state aerobic protocol with oral appliance use. For most healthy individuals, it takes approximately 2 to 3 min to attain a steady‐state heart rate; thus, for the purposes of this study, the goal was to bring subjects to steady state and assess impact on performance with and without an oral appliance (Kenney, Wilmore, & Costill, 2015). The outcomes of the respiratory rate and ventilation for this current study (respiratory rate, 8.8% difference between no OA and OA conditions) are in line with the Garner study, which cited 30.63 BPM for a no OA condition and 27.92 BPM for a boil and bite oral appliance (9.25% difference) and Bourdin et al. citing a 5% improvement (34.2 BPM without OAand 32.5 BPM with OA) (Bourdin et al., 2006; Garner, 2015). In addition, the mean volume of oxygen consumption was the same despite the lowered respiratory rate, which indicates that oxygen consumption was not compromised by the mouthpiece, nor was it lower despite lowered respiratory rate. Although during the first 7 min of the run there were decreases in carbon dioxide with OA use versus no OA use, these differences were not significant and dissipated during the last 3 min. Greater carbon dioxide with no OA use conflicts with a prior study, which found a significant increase in carbon dioxide with OA use, yet supports a later study finding no differences in carbon dioxide with oral appliance use (Garner, 2015; Garner, Dudgeon, Scheett, & McDivitt, 2011).

The primary purpose of this study was to determine the effects of an oral appliance designed to act on the genioglossus and thereby affect respiratory parameters during exercise. The outcomes from this study demonstrate that the use of this oral appliance does positively affect respiratory parameters (lowered respiratory rate and ventilation and no lowering of oxygen intake) and thereby encourage athletes to use for performance, which may provide a minimal level of protection against damage to the lower dentition during sports and exercise. In addition, the decrease in respiratory rate could affect fatigue and potentially prolong exercise performance, with future studies needed to verify the effect on lowered respiratory rate on performance and recovery parameters. Finally, further studies are need to determine if the decreases in respiratory rate, tongue placement, or jaw position could also explain improved physiological outcomes cited in previous studies such as decrease lactate and/or cortisol (Arent et al., 2010; Dudgeon et al., 2017; Garner, Dudgeon, & McDivitt, 2011; Garner, Dudgeon, Scheett, & McDivitt, 2011; Garner & McDivitt, 2015; Morales et al., 2015).
